# Lutein and Zeaxanthin Isomers Reduce Photoreceptor Degeneration in the* Pde6b*^*rd10*^ Mouse Model of Retinitis Pigmentosa

**DOI:** 10.1155/2018/4374087

**Published:** 2018-12-17

**Authors:** Minzhong Yu, Weiming Yan, Craig Beight

**Affiliations:** ^1^Department of Ophthalmic Research, Cole Eye Institute, Cleveland Clinic Foundation, Cleveland, OH, USA; ^2^Department of Ophthalmology, Cleveland Clinic Lerner College of Medicine of Case Western Reserve University, Cleveland, OH, USA; ^3^Department of Clinical Medicine, Faculty of Aerospace Medicine, Key Laboratory of Aerospace Medicine of the National Education Ministry, Fourth Military University, Xi'an, China; ^4^Louis Stokes Cleveland Veterans Affairs Medical Center, Cleveland, OH, USA

## Abstract

**Purpose:**

Lutein, RR-zeaxanthin, and RS-zeaxanthin (L-Z) are antioxidants which can reduce endoplasmic reticulum stress (ERS) and oxidative stress (OS), and ameliorate neurodegenerative diseases. However, their treatment effect in the* Pde6b*^*rd10*^ (*rd10*) mouse model of retinitis pigmentosa (RP) and the underlying cellular mechanisms have not been studied. ERS is an important factor which causes photoreceptor apoptosis. The aim of the current project is to test the treatment effect of L-Z in* rd10* mice and to investigate the underlying molecular mechanisms of ERS.

**Methods:**

L-Z (Lutemax 2020, 10 mg/kg) diluted in sunflower oil (SFO, 1 mg/ml) or the same volume of SFO was administrated via gavage from postnatal day 6 (P6) to P20 daily in L-Z group (n=5) or SFO group (n=6) of* rd10* mice. At P21, electroretinography (ERG) was performed to show the functional change of retinas. 78 kDa glucose-regulated protein (GRP78) and endoplasmic reticulum protein 29 (ERp29) were tested by western blot and immunostaining.

**Results:**

The ERG amplitudes were larger in the L-Z group than those of the SFO group in all flash luminances of dark-adapted and light-adapted ERG (all* p *< 0.01). Western blot revealed that GRP78 in the retinas of the L-Z group was significantly downregulated compared to that of the SFO group (*p *< 0.01). Meanwhile, the retinal ERp29 protein was significantly upregulated in the L-Z treatment group than that of the SFO group (*p *< 0.01).

**Conclusions:**

L-Z provide protection to the photoreceptors of* rd10* mouse model of RP, which is probably associated with the reduction of ERS.

## 1. Introduction

Retinitis pigmentosa (RP) is an inherited retinal degenerative disease which affects one in 3500-5000 individuals [1]. The common clinical symptoms of RP include night blindness and progressive vision loss from peripheral to central vision due to the degeneration of rod and cone photoreceptors, which could eventually lead to irreversible blindness [2]. However, the exact pathologic mechanism of the photoreceptor death has not been fully understood. It was found that the death of cone as well as rod may be due to OS [3] and ERS [4]. As RP is a kind of disease resulting from mutant genes, increasing knowledge of the causative genes with the associated biochemical pathogenesis have been gained, and gene therapy has emerged as one of the most potential treatments for RP [5, 6]. Recently, the U.S. Food and Drug Administration has approved the first gene therapy for a type of RP, Leber congenital amaurosis, which is caused by a mutant RPE65 gene [7, 8]. However, RP is highly genetically heterogeneous with over 200 mutations linked to more than 60 human genes [9, 10]. Until now, it is still not feasible to correct all RP mutations. Therefore, it is necessary to study pharmacological intervention that targets the common and major cellular signaling pathways of the pathogenesis of RP to control retinal degeneration [11].

Although RP is genetically heterogeneous, apoptosis is widely recognized as the general final pathway for the death of photoreceptors [12]. ERS, caused by excessive misfolded proteins in the ER, could activate the ER unfolded protein response (UPR) and the apoptotic pathways [13–15], which has been associated with neurodegenerative disorders, including Alzheimer's disease and Parkinson's disease [16, 17]. These diseases cause the accumulation of misfolded proteins and ERS, which could eventually lead to the apoptosis of neuronal cells [18]. In addition, a lot of studies provide evidence that ERS is present in many types of photoreceptor degenerations, such as light-induced retinopathy [19], experimental retinal detachment [20], achromatopsia, and RP [21]. In addition, the interventions that ameliorate ERS render protection to photoreceptors and reduce the rate of photoreceptor death [22].

L-Z, the two major xanthophylls, are the oxygenated forms of carotenoids [23] that are usually accumulated in the central retina of human eyes [24], with the protective effect in human and animal models of retinal degeneration [25–31]. However, the mechanism of L-Z in RP has not been clearly elucidated. The main mechanism of the protective effect of L-Z is attributed to the antioxidant ability [32, 33], while the reduction of ERS might be another related mechanism. We previously showed that L-Z ameliorated the ERS in the protection against light-induced retinopathy [34]. The* rd10* mouse is a RP model with clear characteristics [35–38], which carries a missense point mutation in exon 13 of the rod phosphodiesterase 6B (*Pde6b*) gene [36, 37]. The mutations in the same gene also cause human RP [39, 40]. In the present study, the protective effect of L-Z on the retinas of* rd10* mice was investigated. Furthermore, the involvement of ERS was also explored to explain the protective effect of L-Z on the retinal degeneration of* rd10* retinas.

## 2. Materials and Methods

### 2.1. Animals and Experimental Design

The breeding pairs of* rd10* mice (Stock Number: 004297) were purchased from the Jackson Laboratory (Bar Harbor, ME). All animal procedures were approved by the Institutional Animal Care and Use Committee of the Cleveland Clinic Foundation and were performed in accordance with the Association for Research in Vision and Ophthalmology Statement for the Use of Animals in Ophthalmic and Vision Research.

L-Z (Lutemax 2020, 10 mg/kg of body weight, OmniActive Health Technologies, Mumbai, India) dissolved in SFO (1 mg/ml, W530285, Sigma, St. Louis, MO) or the same volume of SFO was administered by daily oral gavage from P6 to P20 to* rd10* mice in L-Z treatment group (n=5) or SFO group (n=6), respectively. This dose of L-Z was used based on our preliminary data of different doses (data not shown), which is the highest dose without significant adverse effect on the growth of body weight. The mice in each litter were randomly assigned to the L-Z group and the SFO group. At P21, ERG was performed to show the change of retinal function. The order of the mice in the same litter for ERG test was random. 78 kDa glucose-regulated protein (GRP78) and endoplasmic reticulum protein 29 (ERp29) were tested by western blot and immunostaining. The number of the animals used was conservative enough for obtaining the statistical power of 0.8 or higher.

### 2.2. Electroretinography (ERG)

Before ERG test, the mice were taken out from a cabinet in which the mice were kept for overnight dark adaptation, and were anesthetized with ketamine (80 mg/kg) and xylazine (16 mg/kg) dissolved in saline solution. The pupils were dilated with 1% cyclopentolate HCl, 2.5% phenylephrine HCl, and 1% tropicamide. The corneal surface was anesthetized with 0.5% proparacaine HCl. During the ERG test, the mouse was placed onto a platform with a heating pad in a grounded Faraday cage to keep the body temperature of the anesthetized mouse at 37°C and to reduce electromagnetic interference. The amplitudes of a-wave and b-wave were tested in a series of flash luminances to obtain the luminance-response functions of dark-adapted and light-adapted ERG. In dark-adapted session of ERG test, the flash luminance ranged from -3.6 to 2.1 log cd s/m^2^. In light-adapted session of ERG test, the flash luminance ranged from -0.8 to 1.9 log cd s/m^2^. The light-adaptation time was 7 minutes before the first light-adapted ERG was recorded. The a-wave and b-wave amplitudes were used for the evaluation of the retinal function. The a-wave amplitude was measured from the baseline to the amplitude at 8 ms after the onset of flash stimulation. The b-wave amplitude was measured from the a-wave trough to the b-wave peak, using the published procedure [34, 41–46].

### 2.3. Western Blotting

The same protocol in our previous study [34] was used. Briefly, the retinas were extracted from mouse eyeballs with a cut on the cornea, and were homogenized. The proteins were obtained in the supernatant after centrifugation of the lysates at 16,000 x g for 25 minutes at 4°C and measured by Pierce 660 nm Protein Assay Reagent (Thermo Scientific, Rockford, IL). Equal amounts of protein (15 *μ*g) of each extract in Laemmli Sample buffer were heated for 7 minutes and then electrophoresed on 8-16% gradient sodium dodecyl sulfate- (SDS-) polyacrylamide gel. Afterwards, the proteins were transferred to a polyvinylidene difluoride (PVDF) membrane. The membrane was blocked and then incubated with primary antibodies (anti-GRP78/BiP, 1:300, ab21685 and anti-ERp29, 1:2500, ab11420, Abcam, Cambridge, MA) in Tris buffered saline with Tween 20 (TBST) for 16 hours at 4°C. After washing 3 times with TBST, the membrane was incubated with secondary antibody goat-anti-rabbit Immunoglobulin G- (IgG-) horseradish peroxidase (HRP) (1:7000, sc-2004, Santa Cruz, CA) in TBST for 1 hour at 25°C. The membrane was then washed 3 times with TBST. Finally, the antigen-antibody complexes were detected by the enhanced chemilu minescence-2 (ECL-2, Thermo Scientific, Rockford, CA).

### 2.4. Immunostaining

The eyeballs were harvested and fixed in 4% paraformaldehyde (PFA) with 1X phosphate buffer saline (PBS, pH 7.4) for 20 minutes and then a hole was punched on the cornea. The eyes were put back to the 4% PFA fixative for additional 4 hours. Afterwards, the eyeballs were immersed in three graded sucrose solutions in 1X PBS in the following order/time: 10% for 1 hour, 20% for 1 hour, and 30% overnight. The eyeballs were then embedded in optimum temperature cutting compound (OCT), flash frozen on dry ice, and stored in −80°C freezer for at least overnight. The eyeballs in OCT were sectioned with a cryostat (Leica CM 1850, Buffalo Grove, IL) to 8 micron sections. Protein expression* in situ* was tested by immunofluorescence staining. The sections were incubated in 1X PBS containing 5% normal goat serum, 1% bovine serum albumin (BSA), and 0.5% Triton X-100 for 1 hour to block nonspecific binding, followed by incubation with primary antibodies (anti-GRP78/BiP, 1:400, ab21685 and anti-ERp29, 1:500, ab11420, Abcam, Cambridge, MA) overnight at 4°C. After three washes with 1X PBS, the sections of retinas were incubated with secondary antibody goat anti-rabbit IgG H&L Alexa Fluo® 555 (1:600, ab150086, Abcam, Cambridge, MA) for 2 hours at room temperature. After washing with 1X PBS, sections were mounted with VECTASHIELD mounting medium with 4′,6-diamidino-2-phenylindole (DAPI, Vector Laboratories, Burlingame, CA) and examined under a fluorescent microscope (BX61, Olympus, Tokyo, Japan).

### 2.5. Measurement of ONL Thickness

The DAPI-stained images obtained in the above procedure were used for the measurement of the thickness of outer nuclear layer (ONL). The locations for the measurement of ONL thickness were at 200 *μ*m from the edges of the optic disc on both sides of the optic nerve head. ImageJ 1.48v software (National Institutes of Health, Bethesda, MD) was used for the measurement. In each retina, four sections were used to obtain the average of ONL thickness.

### 2.6. Statistical Analysis

For the analysis of ERG data, two-way repeated measures analysis of variance (ANOVA) was performed. For the analysis of the other data, one-way ANOVA was performed.

## 3. Results

### 3.1. The Effect of L-Z on Retinal Function of rd10 Mice

**Figure 1:**
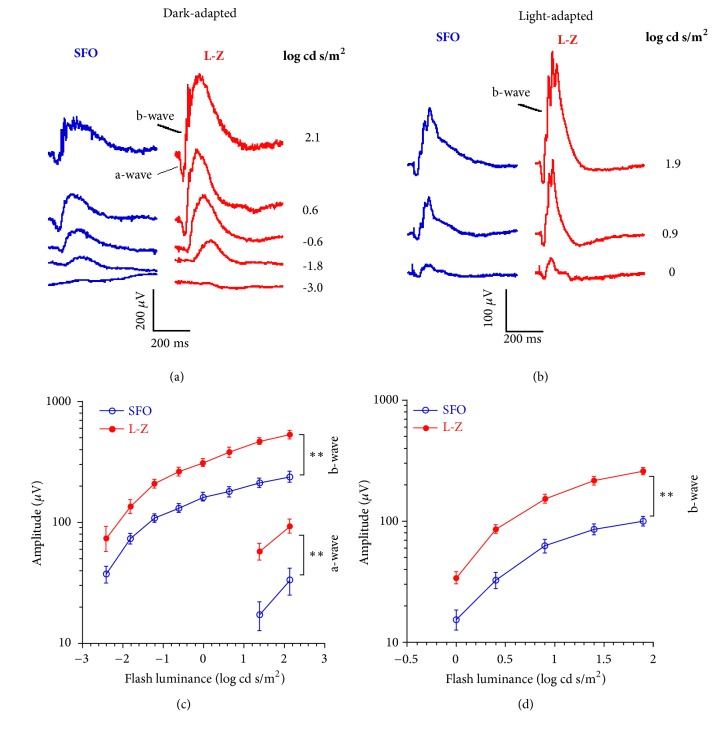
**ERG results obtained from* rd10 *mice at P21 treated with L-Z (n = 5) or SFO (n = 6)**. (a) Typical dark-adapted ERG waveforms from both groups at P21. (b) Typical light-adapted ERG waveforms from both groups at P21. (c) Luminance-response curves of a-wave and b-wave in dark-adapted ERG at P21. (d) Luminance-response curves of b-wave in light-adapted ERG at P21. Under both dark-adapted and light-adapted conditions, the ERG a-wave and b-wave amplitudes were significantly higher in the L-Z treatment group than those in the SFO group at P21 under all of the luminances tested. *∗∗ p* < 0.01.

The retinal function of mice was tested by ERG (Figure 1). The ERG waveform typically consists of a-wave followed by b-wave. In dark-adapted ERG, the a-wave is the response from rod or rod/cone photoreceptors, and the b-wave is mainly from bipolar cells of the rod or rod/cone pathways, which is also affected by the output of rod or rod/cone photoreceptors. The b-wave of the light-adapted ERG is from bipolar cells of the cone pathway, which is also affected by the output of cone photoreceptors. Our ERG data showed that the a-wave and b-wave amplitudes of the dark-adapted ERG were significantly larger in the L-Z treatment group than those in the SFO group under the flash luminances we used (all* p* < 0.01). In addition, the b-wave amplitudes of the light-adapted ERG of the L-Z treatment group were significantly higher than those in the SFO group under all of the flash luminances tested (all* p* < 0.01).

### 3.2. The Effect of L-Z on ERS in rd10 Mice

**Figure 2:**
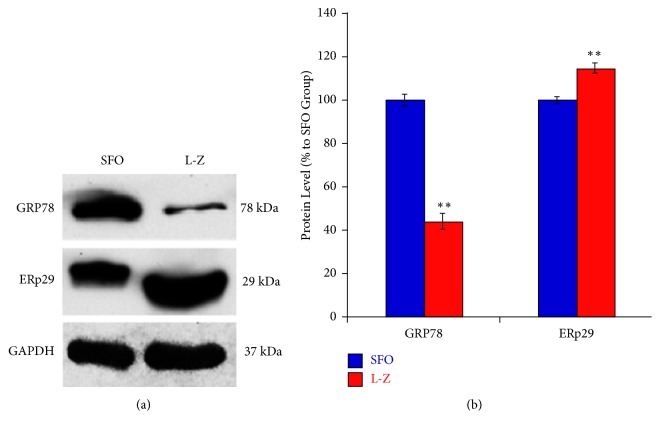
**Western blot results of ER stress protein markers (GRP78 and ERp29) in the L-Z treatment group and the SFO group**. (a) Representative images of western blot for GPR78. (b) Average protein expression of GRP78 (n = 3) in both L-Z-treated and SFO groups. *∗∗ p *< 0.01 versus SFO group. The GRP78 was significantly downregulated in the L-Z group than that of the SFO group, while the ERp29 was significantly upregulated in the L-Z group compared to that of the SFO group.

GRP78 acts as the main ER chaperon of the UPR, while the role of ERp29 has not been clearly elucidated in ERS. They were tested by western blot and immunostaining in this study. Our western blot data (Figure 2) revealed that GRP78 in the retina of the L-Z treatment group was significantly downregulated compared to that in the SFO group (*p *< 0.01). Meanwhile, the retinal ERp29 was significantly upregulated in the L-Z treatment group than that of the SFO group (*p *< 0.01).

**Figure 3:**
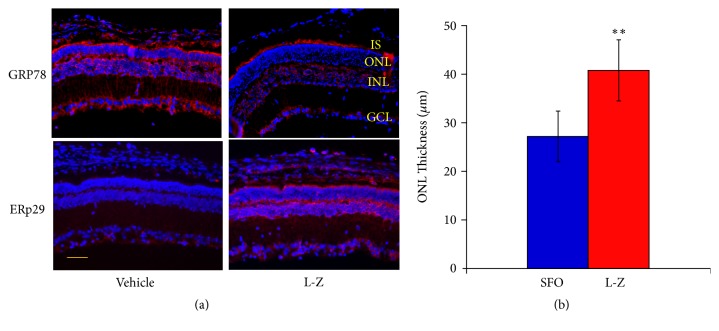
**Immunostaining results of GRP78 and ERp29 in the L-Z treatment group and the SFO group**. (a) Representative images (20x) of retinal immunostaining for GRP78 and ERp29. Scale bar indicates 50 *μ*m. The GRP78 was markedly downregulated in several retinal layers in the L-Z group than that of the SFO group, while the ERp29 was markedly upregulated in some retinal layers in the L-Z group compared to that of the SFO group. (b) The ONL thickness (mean ± SD) based on the DAPI staining in these images was significantly increased in the L-Z group than in the SFO group. IS: inner segment; ONL: outer nuclear layer; INL: inner nuclear layer; GCL: ganglion cell layer. n = 3. *∗∗ p* < 0.01 versus SFO group.

Immunostaining of GRP78 showed that GRP78 protein was expressed mainly in the inner segment (IS), inner nuclear layer (INL), outer plexiform layer (OPL) and ganglion cell layer (GCL) in the SFO group. This result is consistent with the finding of another study [47] and our previous studies [34, 41, 42]. On the other hand, there was a marked downregulation of GRP78 in the above retinal layers in the L-Z treatment group compared to the SFO group (Figure 3). The immunostaining of ERp29 revealed that there was a weak expression of ERp29 presented only in the GCL of the SFO group. However, ERp29 expression was upregulated in IS, OPL, INL and GCL in the retina of the L-Z group compared to the SFO group (Figure 3).

### 3.3. The Effect of L-Z on ONL Thickness in rd10 Mice

The ONL thickness (mean ± SD) was measured in the DAPI staining of immunostaining images, which showed significant increase of ONL thickness in the L-Z group than in the SFO group (*p* < 0.01).

## 4. Discussion

The present study revealed the protective effect of L-Z on photoreceptor degeneration of* rd10* mice, an animal model of retinitis pigmentosa. The ERG results in our study showed that L-Z treatment improved rod and cone functions in* rd10* mice, rendering protective effect on both rod and cone photoreceptors.

*rd10* mice have long been established as an animal model of RP in several studies. It was observed that at 2 weeks of age, the photoreceptors of* rd10* mice already show minor photoreceptor degeneration [48]. By 8 weeks of age, most of the photoreceptors disappear [49]. The progressive photoreceptor degeneration in this strain of mice is attributed to a homozygous point mutation in* Pde6b* gene [36, 37]. Some mutations in* Pde6b* gene have also been found in human RP patients [50, 51]. The photoreceptor degeneration in* rd10* mice leads to deterioration of the retinal function. The a-wave and b-wave of ERG are still detectable at 3-week-old mice, but these components are not recordable at the age of 5 weeks [52].

The antioxidants used for the treatment in this study are L-Z, which can neutralize reactive oxygen species to counter oxidative damage in retinal cells in many eye disorders [23, 53–56]. In addition, our previous study showed that L-Z reduce ERS in light-induced retinopathy [34].

Endoplasmic reticulum (ER) is the organelle for protein folding and assembly. When the cellular homeostasis is interfered, the protein misfolding or unfolding induces ERS and activates the UPR, which can reduce ERS by increasing ER protein folding capacity, increasing degradation of misfolded proteins, and suppressing the translation of proteins. However, under persistent ERS, the UPR signaling can cause cell death by activating the intrinsic pathway of apoptosis [57–61]. ERS is associated with many retinal diseases, including RP. The amelioration of ERS can reduce photoreceptor degeneration in* rd10* mice [41, 42, 62, 63]. In this study, our result illustrated that administration of L-Z reduced the ERS, which was indicated by the downregulation of GRP78, a marker of ERS [64–66].

While our GRP78 test verified that L-Z reduced the ERS, we are also interested in the expression of endoplasmic reticulum protein 29 (ERp29) which was investigated in recent retinal studies [67, 68]. ERp29, ubiquitously expressed in the ER membrane among various tissues and cell types, is an ER luminal protein in all mammals [69, 70]. It plays a pivotal role in modulating the folding and transportation of proteins during ERS [67]. GRP78 is one of the main targets of ERp29 [69]. In addition, ERp29 could also regulate other proteins, such as p38, p58^IPK^ and heat shock protein 27 (Hsp27), for cell survival or apoptosis during ERS [71, 72].

According to previous studies, the change of ERp29 level has at least two phases of either upregulation or downregulation during ERS [68, 73]. Zhang et al. reported that ERp29 expression is beneficial to cellular viability. Overexpression of ERp29 in ARPE-19 cell line with cigarette smoke extract- (CSE-) triggered ERS reduced the number of apoptotic cells, while the inhibition of ERp29 upregulated CSE-triggered CCAAT/enhancer-binding protein homologous protein (CHOP) expression and induced cell apoptosis [68]. The ERS induced by 6-hour, 24-hour and 10-day CSE administration upregulated ERp29. However, during 3-week CSE administration, the expression of ERp29 was downregulated while the GRP78 was still upregulated. Furthermore, a peak level of ERp29 expression was induced by a medium level of CSE in their study [68]. In the study of Park et al., ERp29 was downregulated 1 day after spinal injury and upregulated around 7 days after spinal injury [73]. Therefore, it is possible that the direction of the change of ERp29 level is associated with the level or duration of ERS. In our study, ERp29 was upregulated while the GRP78 was downregulated after the L-Z treatment, which implies that ERp29 may reduce ERS and protect retinal cells. Our result of the SFO group is consistent with the result in the 3-week CSE-induced ERS experiment of Zhang et al. [68]. In the SFO group of* rd10* retinas, it is possible that there is long-term ERS. It is not surprising that the high level of GRP78 and the low level of ERp29 were shown in this group. After L-Z treatment, the oxidative stress was reduced in the* rd10* retinas, which reduced the ERS indicated by the reduction of GRP78 level [74–78]. In the meantime, the ERp29 level was increased due to the change of ERS level, which in turn downregulated the expression of GRP78 and finally reduced the photoreceptor apoptosis.

## 5. Conclusions

In conclusion, L-Z prevent photoreceptors from degeneration in* rd10* retinas, which is possibly associated with the reduction of ERS. Future studies are necessary to clarify the details of the cellular pathways in which L-Z provide the protective effect against retinal degeneration. Our study reveals the possible target of ERS in photoreceptors for the treatment of retinal degenerations.

## Data Availability

The data used to support the findings of this study are available from the corresponding author upon request.
